# Breaking with the status quo in end‐of‐life care through de‐implementation

**DOI:** 10.1111/joim.20086

**Published:** 2025-04-17

**Authors:** Chetna Malhotra, Ellie Bostwick Andres

**Affiliations:** ^1^ Lien Centre for Palliative Care Duke‐NUS Medical School Singapore Singapore

**Keywords:** de‐implementation, end‐of‐life care, low‐value care, patient‐centred care, terminal care

## Abstract

This paper addresses the challenge of de‐implementing low‐value care practices in the end‐of‐life (EOL) context, where burdensome interventions often offer marginal life‐extending benefits, incur substantial costs and diminish quality of life. We examine the complexities involved in discontinuing such practices, including clinician biases, institutional cultures favouring aggressive interventions and communication barriers among healthcare providers, patients and families. We discuss how de‐implementation at the EOL is unique from other contexts, prioritizing patient‐centred care rather than cost reduction. Effective communication and support for patients, families and clinicians is essential, as de‐implementation often represents a shift towards what patients and families value. Our review of existing evidence underscores the need for the development and evaluation of de‐implementation strategies tailored to EOL care, as described. De‐implementation at the EOL requires sensitivity to the complex, emotional nature of EOL care and provides a unique opportunity to integrate palliative care approaches and improve overall EOL care quality.

## Graphical abstract

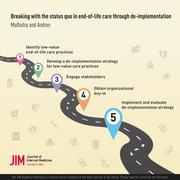

*Mrs T is a 93‐year‐old woman with advanced dementia. For the past 18 months, her inability to eat has been managed with a nasogastric feeding tube. She has experienced recurrent urinary tract infections, leading to hospitalizations involving intravenous antibiotics and physical restraints, which have further deteriorated her physical and cognitive health. Mrs T's daughter is distressed by her mother's ongoing medical challenges and believes the frequent hospitalizations and aggressive treatments are inconsistent with the life Mrs T would have chosen for herself*.

*What strategies can healthcare organizations use to de‐implement low‐value care practices and improve care experiences for patients and families in similar situations?*

## Introduction

In the realm of serious illness, many patients undergo interventions that may marginally prolong life but often sacrifice quality of life and entail significant costs [1, 2]. These interventions, categorized as ‘low‐value care’, often involve complex procedures, frequent hospitalizations and intense medical management, leading to considerable discomfort, reduced functional ability and overall decreased well‐being and calling into question the efficiency and effectiveness of current end‐of‐life (EOL) care practices. From the perspective of patients and families, such low‐value care is often misaligned with their personal values and EOL care goals [3]. As with Mrs T, this misalignment can cause substantial distress and dissatisfaction with care [4]. Moreover, studies reveal that low‐value care accounts for up to 30% of medical spending [5, 6, 7].

The Lancet Commission on the Value of Death highlights low‐value care as a widespread issue for seriously ill patients [8]. A systematic review of 38 studies found one third of older patients received hospital interventions classified as low‐value in their last 6 months of life, including dialysis, radiotherapy, transfusions, antibiotics, life support treatments and unnecessary, often invasive investigations [9]. Many EOL care practices, such as the use of antidepressants and feeding tubes in dementia, are identified as low‐value care by *Choosing Wisely*, an international physician‐led campaign to de‐implement such care practices [5, 10–12].

This paper explores several key aspects of de‐implementation in EOL care, including the challenges of de‐implementing low‐value care in the EOL context, how de‐implementation at the EOL differs from other healthcare contexts and existing evidence on de‐implementation strategies. Finally, for healthcare administrators and researchers, we include guidance for successfully conducting de‐implementation within healthcare organizations.

### The challenges of de‐implementing low‐value care in the EOL context

Despite evidence indicating certain practices are low‐value at the EOL, clinicians may fear providing too little treatment to critically ill patients rather than too much [13], relying on heuristics or unconscious mental shortcuts to make swift decisions, particularly in acute settings where they may have no context for the patient and family's preferences [14]. Additionally, clinicians frequently lack the time for shared decision‐making and may struggle to convey complex information about treatments and prognosis, often due to a reluctance to undermine patients’ and families’ hope [15]. Even in situations where patients documented a preference for ‘comfort care’, there could be difficulty discerning which treatment pathway constitutes ‘comfort care’ [16]. The uncertainty in illness trajectories and clinician optimism in predicting patient outcomes further fuel low‐value practices.

Patients and families are especially vulnerable in the context of EOL care and may defer to their clinicians’ recommendations and be reluctant to question medical advice. This power imbalance hinders open communication, impedes shared decision‐making and enables continued low‐value care practices [14]. Many terminally ill patients lack the capacity to make decisions and families, driven by pre‐death grief, may be willing to expend more for treatments offering marginal improvements in life extension due to their reluctance to let go of their loved ones [3, 17, 18].

Hospital cultures that default to high‐intensity interventions [19, 20], combined with financial incentives that promote the use of more services and treatments [21] and the siloed nature of specialities that limit interdepartmental communication [22], further perpetuate low‐value care and can even undermine an individual clinician's efforts to de‐implement such practices. From a policy perspective, discussions about reducing low‐value care often raise concerns about care rationing and ageism [23]. Some argue certain interventions, regardless of their actual benefits, provide ‘hope’ to patients and families, suggesting the value of hope should be considered in health technology assessments [24].

In summary, low‐value care at the EOL is influenced by various factors, including individual (patient, family and clinician‐related), organizational, financial and policy‐related elements. Therefore, strategies to de‐implement low‐value care at the EOL should address multiple, if not all, of these factors.

### De‐implementation at the EOL differs from other healthcare contexts

As highlighted above, the distinction between low‐value and comfort care or symptom management at the EOL is often blurred. With a high degree of uncertainty surrounding benefits and risks and a lack of clear, consensus‐driven guidelines addressing every EOL scenario, decisions frequently depend on subjective judgements shaped by cultural and societal perceptions and beliefs [25].

Additionally, given the sensitive nature of EOL care, de‐implementation efforts risk being misinterpreted as rationing, unfair or culturally insensitive [26]. For example, patients and families accustomed to a comprehensive medication regimen might resist efforts to reduce ‘unnecessary’ medications at the EOL, which could affect the patient–provider relationship [27]. Similarly, minority groups may view service limitations with scepticism due to historical inequities in palliative and hospice care [28].

Hence, unlike other healthcare contexts—where the focus is on removing, reducing or restricting low‐value care—EOL care requires a more nuanced approach. Thus, messaging is key. The previous research warns against the use of terms such as ‘unnecessary care’ and a focus on cost‐cutting, as these can erode clinician engagement and patient trust [5, 29]. Instead, every therapeutic intervention should be evaluated to determine whether it promotes the agreed‐upon EOL care goals [30]. For example, unlike many *Choosing Wisely* recommendations that call for the elimination of particular tests or practices, one of the Australasian College for Emergency Medicine *Choosing Wisely* recommendations advises that, for emergency department patients approaching EOL, clinicians, patients and families should share a common understanding of the goals of care. Although reducing low‐value care is still the objective, patient‐centred EOL care is the priority.

Effective communication with patients and caregivers is crucial, and discussions about de‐implementation must be handled with sensitivity [31]. Primary care providers and family doctors are especially suited to effectively take on such difficult conversations with compassion, given their longitudinal relationships with patients and their families, awareness of preferences and established trust. By leveraging their continuity of care, they can address concerns, provide reassurance and facilitate shared decision‐making. Should patients and families still value high‐intensity treatments, clinicians should remember that there is no one‐size‐fits‐all solution; EOL care is inherently individualized.

Finally, EOL care also represents a context in which low‐value care is common, yet palliative care remains underutilized [32]. Thus, de‐implementation presents an opportunity to replace low‐value practices with palliative care, a high‐value alternative. Such an approach shifts the focus from abandoning care to prioritizing care that aims to enhance quality of life.

### De‐implementation strategies in EOL settings

Existing literature on de‐implementation within EOL care has primarily focused on identifying facilitators and barriers for de‐implementation [7, 26]. We reviewed published reviews to examine the evidence for de‐implementation strategies used in the EOL context (details in appendix). We found that included studies had examined strategies targeting providers, patients and organizational approaches, often addressing multiple groups simultaneously [33, 34, 35, 36]. These strategies are frequently coupled with implementation efforts, such as palliative care, designed to replace low‐value practices [37, 38, 39, 40, 41, 42].

Provider‐directed interventions include education, audit and feedback, clinical guidelines, decision support and the use of electronic medical records (EMR) to support decision‐making at the point of care [13, 29, 33, 43]. There was limited evidence that simply educating clinicians reduced life‐sustaining treatments at the end of life [44]. A review of strategies to reduce ICU admissions among terminally ill patients found limited conclusive evidence for a particular strategy but that advised goals of care conversations should be initiated in the pre‐hospital and outpatient settings and extend into the hospital setting in advance of ICU admission to further guide whether admission is consistent with a patient's values and goals of care [45].

Patient‐ and family‐directed interventions include patient education materials and decision‐support tools [33, 46]. A number of patient and family decision aids have been developed to support decisions involving care considered low‐value, such as feeding tubes in advanced dementia or dialysis in end‐stage kidney disease [47]. Such decision aids have been shown to improve patient and family understanding, though none have shown effectiveness in reducing low‐value practices to date.

A combination of strategies targeting both patients/families and providers have been used to encourage communication and shared decision‐making around low‐value care at the EOL. A Cochrane review of interventions to improve communication between patients/families and providers about EOL care found no conclusive evidence [48]. A review of 5 RCTs and 31 observational studies evaluating interventions to reduce aggressive EOL care noted that documentation of patient/family‐provider discussions was associated with reduced use of aggressive measures [49]. However, a review of advance care planning (ACP) interventions found little evidence that ACP reduced healthcare use [50]. Nonetheless, a meta‐analysis examining the association between ACP and aggressive or comfort‐focused care at the EOL found that ACPs with a communication focus were associated with reduced odds of hospital admission and increased use of hospice at the EOL [51].

Organization‐directed strategies are commonly implemented at the hospital or long‐term care facility level, where the greatest reductions in low‐value care have been documented to date [34]. Strategies such as pharmacist review of medication regimens and multidisciplinary team meetings successfully reduced polypharmacy and the use of psychotropic drugs, respectively, in long‐term care facilities. Likewise, organization‐directed staff education and multi‐component interventions, including training, education and policy adoption, led to reduced restraint use among older adults with dementia [52, 53]. Another review found that hospital‐initiated, multi‐component, multidisciplinary deprescribing interventions, which include medication reviews and care coordination, were effective in reducing polypharmacy in older terminally ill patients in the short term, though evidence of long‐term impact was lacking [54]. A scoping review of initiatives to reduce inappropriate or non‐beneficial hospital admissions and bed days at the end of life reported limited evidence of effectiveness, with most studies failing to identify whether fewer admissions were concordant with patient values and goals of care [55].

Overall, research on the effectiveness of de‐implementation strategies in EOL care is limited and inconsistent. Organization‐wide strategies and promotion of goals‐of‐care discussions coupled with support for patients and families show more promise compared to interventions that focus solely on clinician or patient education. The limited existing evidence underscores the need for developing de‐implementation strategies tailored to EOL care [56, 57].

### How to conduct de‐implementation in EOL contexts

**Fig. 1:**
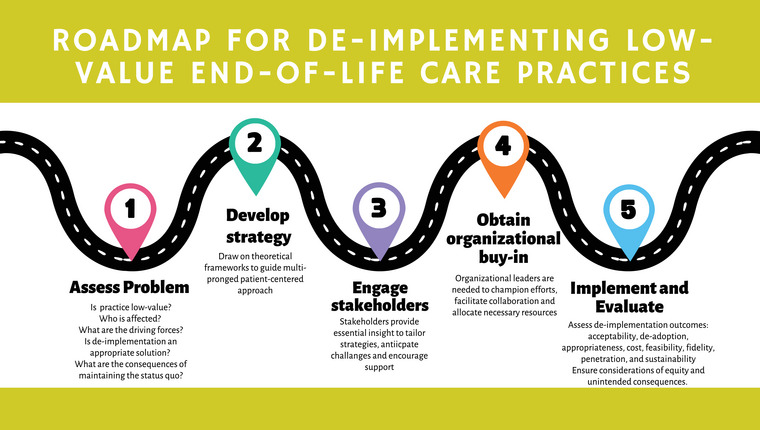
De‐implementing low‐value end‐of‐life care practices. Source: De‐implementation outcomes from Ref. [26].

For healthcare administrators and researchers looking to de‐implement low‐value care within their organizations, we recommend a five‐step process (Fig. 1).

*First*, it is important to carefully assess whether the EOL care practice is low‐value and de‐implementation is the right solution. With its potential to reduce costs and improve quality, investigators may be tempted to rush into de‐implementation [58]. However, thoroughly articulating the problem and understanding its scope are essential first steps. Identify who is affected and in what way, review the evidence and guidelines claiming the practice is low‐value, examine the factors driving the practice and its effects [29] and consider the consequences of maintaining the status quo [10, 13, 59, 60]. Taken together, this information will inform the assessment of the feasibility of de‐implementation and help calibrate the approach [13, 26, 29, 61].

*Second*, a holistic multi‐stakeholder approach to de‐implementation must be considered. Simply sharing evidence of a low‐value practice is unlikely to prompt clinicians to abandon it. Further, a clinician's efforts to discontinue low‐value care practices may be hindered if they are not in sync with the organization's culture and policy‐driven incentives. Thus, a broader approach targeting both individual behaviours and organizational practices is required [62].

Theoretical frameworks can guide the development of the multi‐pronged strategy. Three scoping reviews of theoretical frameworks used in de‐implementation research provide a good reference for framework selection and attest to its nascent state and growing interest [6, 63, 64]. A common finding in all three of the scoping reviews was the unique importance, relative to implementation, of considering patient preferences, which the authors suggest are more commonly accounted for in recent studies [6, 63, 64]. Compared to implementing an evidence‐based standard of care, de‐implementation necessitates special consideration for how patients and other stakeholders will respond [26].

To date, most de‐implementation research has applied *determinant* frameworks, such as the Theoretical Domains Framework [65] and the Consolidated Framework for Implementation Research [64, 66], which consider contextual factors influencing successful de‐implementation. *Process* frameworks, such as the classic theory of planned behaviour [67], guide and describe the steps involved in initiating de‐implementation and are the second most commonly identified in existing de‐implementation studies [43]. An *evaluation* model, such as reach, effectiveness, adoption, implementation and maintenance [68], might be appropriate for determining whether de‐implementation was successful. The synthesis model for the process of de‐adoption developed by Niven et al. specifically considers de‐implementation, combining the three types of research inquiry addressed in the above models [69]. It adapts the Knowledge‐to‐Action process framework, conceptualizing de‐implementation as a cycle of assessing the low‐value practice and determinants, intervening to de‐implement the practice, evaluating the process and outcomes and sustaining de‐implementation [69].

*Third*, it is essential to engage and support patients, families and clinicians throughout the de‐implementation process. Clinical champions and patient advocates can provide practical insights into the feasibility and impact of de‐implementation, aiding in the design of more effective and sustainable strategies. Stakeholders may raise concerns or identify potential unintended consequences, which should be addressed and accounted for to achieve successful de‐implementation outcomes [70]. This approach fosters trust and support for the de‐implementation process. Patients and families also need support in adaptive coping strategies as they navigate their grief and emotional stress while foregoing low‐value care.

*Fourth*, securing organizational buy‐in is essential for ensuring leaders are dedicated to driving change. Leaders play a critical role in championing de‐implementation efforts, addressing and overcoming resistance from staff and allocating necessary resources, including staff training and EMR updates. Organizational leaders can facilitate essential training for clinicians in handling the complexities of de‐implementing EOL care with sensitivity, ensuring they can facilitate compassionate care while addressing the concerns of all parties involved. Specifically, training in facilitating goals‐of‐care conversations is critical, as these discussions help align care with patients’ values, manage expectations and foster shared decision‐making.

Organizational endorsement of new practices also helps integrate de‐implementation efforts into the organization's policies and guidelines, fostering a culture of high‐value care. If conflicts or misalignments arise, institutional policies and legal guidance can ensure that care is appropriate, patient‐centred and ethically sound [25]. Additionally, effective EOL care requires seamless coordination among various disciplines, including specialists and palliative care teams. Organizational leaders can facilitate this collaboration.

*Finally*, it is essential that the de‐implementation strategy be implemented with fidelity and evaluated rigorously. Inputs from a range of disciplines, from economics to epidemiology, can ensure a robust evaluation design, implementation and outcome assessment [37]. An *exploratory* de‐implementation study might utilize qualitative methods such as focus groups or stakeholder interviews to assess the feasibility, acceptability and appropriateness of de‐implementation [71]. *Process* evaluations might benefit from a mixed‐methods design, incorporating both qualitative and quantitative outcomes to explore mechanisms of impact, contextual factors and any emergent or complex causal pathways [72]. *Evaluation* studies could use quasi‐experimental and experimental designs to assess the effectiveness and cost‐effectiveness of de‐implementation strategies.

Appropriate outcomes are crucial for demonstrating intervention success and guiding future efforts. Prusaczyk et al. [26] provide guidance on applying eight implementation outcomes in the context of de‐implementation [73]. These include—*acceptability* (stakeholder perception of a practice as unacceptable, and the idea of de‐implementing it is acceptable), *adoption* (intention to stop or reduce a practice, and whether it is de‐implemented equally or targeted to specific groups), *appropriateness* (stakeholder perception of a practice as not appropriate, and the de‐implementation process is appropriate), *cost* (cost of de‐implementing, cost‐effectiveness of de‐implementation, unintended increase in cost), *feasibility* (feasible to de‐implement), *fidelity* (quality of de‐implementation), *penetration* (extent to which a practice is discontinued within a service setting and its subsystems) and *sustainability* (extent to which a practice's discontinuation is maintained) within the specific de‐implementation context.

Additionally, distal outcomes, including de‐implementation efficiency, equity, timeliness, patient‐centredness, unintended consequences and risks or burdens, should be evaluated [26]. Lastly, ethical principles such as informed consent and respect for cultural diversity should always guide de‐implementation research in EOL care.

## Conclusion

Mrs T's experience is emblematic of a widespread issue in EOL care, where low‐value interventions often compromise patient quality of life. Table 1 provides practical insights into how de‐implementation efforts might address one of several low‐value EOL practices plaguing Mrs T—tube feeding in advanced dementia. The challenges in addressing low‐value practices at the EOL are multifaceted, involving clinician biases, hospital culture, financial incentives and communication barriers. To effectively de‐implement such practices, healthcare organizations must adopt a holistic approach that targets these various factors.

**Table 1:** Application of de‐implementation process in context of tube feeding for individuals with advanced dementia.

Steps	Application: Tube feeding for individuals with advanced dementia
1. Assess problem	· Review guidelines: The American Geriatrics Society guidelines discourage tube feeding among persons with advanced dementia as they have not been shown to extend life and commonly reduce quality of life [74] · Examine prevalence of low‐value practice in your setting: 16% of older adults with advanced dementia in Singapore [75] and 34% of persons with advanced dementia in US nursing homes use feeding tubes [76] · Assess what drives the practice: Most feeding tubes are initiated during hospital admissions [77]. Studies show use of feeding tubes is driven by clinician recommendations and fears among family members and carers [78]
2. Develop strategy	· Consider a theoretical framework (e.g., theory of planned behaviour) to tailor the de‐implementation strategy to target both individual behaviours (carer and provider) and organizational policies [67] · Examples of strategy components: ○ Carer education about alternatives to tube feeding; decision aids to support decision‐making; provision of professional and lay support for careful hand feeding ○ Provider education and training; provider EMR prompts for goals of care discussions ○ Organizational policy adoption; interdepartmental collaboration with involvement of palliative care team and nutritionists
3. Engage stakeholders	· Obtain feedback on overall strategy and its individual components, including decision aids and training materials by all stakeholders—carers, persons with mild dementia and providers—to inform its acceptability and appropriateness. Revise strategy to address key concerns. The process should be iterative, continuing throughout the de‐implementation process
4. Obtain organizational buy‐in	· Endorsement of clinical recommendations to prioritize careful hand feeding for persons with dementia at the organization level can facilitate necessary supports, including ○ Provider training on how to engage in goals of care conversations ○ Structural changes and procedural workflows to facilitate collaborations with nutritionists and palliative care teams ○ IT team support to integrate EMR prompts for goals of care discussions based on pre‐defined criteria
5. Implement and evaluate	· Implement strategies and consider a mixed‐methods approach to evaluate both process and impact outcomes ○ Outcomes should measure de‐implementation success, as well as patient, caregiver and clinician experiences and healthcare utilization outcomes, with considerations of equity, patient‐centredness and any unintended consequences ○ Study design would vary based on number of organizations involved and pragmatic considerations

Abbreviation: EMR, electronic medical records.

## De‐implementation strategues in EOL settings

The de‐implementation process requires sensitivity to the complex, emotional nature of EOL care and an awareness of the cultural and historical contexts in which care decisions are made. The emphasis must be on prioritizing patient‐centred care, with a focus on effective communication and support for patients, families and clinicians, since often, de‐implementation represents a shift towards what patients and families value. De‐implementation also provides an opportunity to integrate palliative care approaches that prioritize comfort and align with patients’ values and goals, thus promoting a care model that ensures a dignified and compassionate EOL experience for all patients (Supporting Information).

## Funding information

This work was supported by the National Medical Research Council (MOH‐001031, MOH‐HCSAINV21jun‐0002).

## Conflict of interest statement

The authors declare no conflicts of interest.

## Supporting information




**Supplementary Material**: Search Strategy
