# Supplementary material for: Breaking with the status quo in end‐of‐life care through de‐implementation
Source: J Intern Med. 2025 Apr 17;298(2):97–106. doi: 10.1111/joim.20086 (PMC12239056; doi:10.1111/joim.20086)
Supplement: Supplementary file 1 — Supplementary Material: Search Strategy [file JOIM-298-97-s001.docx]

SUPPLEMENTARY MATERIAL

Search Strategy

1. PubMed:

("reduc*"[Title/Abstract] OR "de-implement"[Title/Abstract] OR "de adopt*"[Title/Abstract] OR "discontinu*"[Title/Abstract] OR "optimiz*"[Title/Abstract] OR "avoid"[Title/Abstract] OR "decreas*"[Title/Abstract] OR "minimis*"[Title/Abstract]) AND ("unnecessary care"[Title/Abstract] OR "low-value"[Title/Abstract] OR "futil*"[Title/Abstract] OR "overtreat*"[Title/Abstract] OR "critical care"[Title/Abstract] OR "aggressive care"[Title/Abstract] OR "aggressive treatment"[Title/Abstract] OR "intensive care"[Title/Abstract]) AND ("end of life"[Title/Abstract] OR "advanced dementia"[Title/Abstract] OR "advanced cancer"[Title/Abstract] OR "end stage"[Title/Abstract] OR "life limit*"[Title/Abstract] OR "terminal"[Title/Abstract] OR "dying"[Title/Abstract] OR "advanced disease"[Title/Abstract] OR "end organ"[Title/Abstract]) AND (review[Filter] OR systematicreview[Filter]) AND (english[Filter])
